# ATR promotes cilia signalling: links to developmental impacts

**DOI:** 10.1093/hmg/ddw034

**Published:** 2016-02-11

**Authors:** Tom Stiff, Teresa Casar Tena, Mark O'Driscoll, Penny A. Jeggo, Melanie Philipp

**Affiliations:** 1 Double Strand Break Repair Laboratory and,; 2 Human DNA Damage Response Disorders Group, Genome Damage and Stability Centre, University of Sussex, BrightonBN1 9RQ, UK and; 3 Institute for Biochemistry and Molecular Biology, Ulm University, 89081 Ulm, Germany

## Abstract

Mutations in *ATR* (ataxia telangiectasia and RAD3-related) cause Seckel syndrome (ATR-SS), a microcephalic primordial dwarfism disorder. Hitherto, the clinical manifestation of ATR deficiency has been attributed to its canonical role in DNA damage response signalling following replication fork stalling/collapse. Here, we show that ATR regulates cilia-dependent signalling in a manner that can be uncoupled from its function during replication. ATR-depleted or patient-derived ATR-SS cells form cilia of slightly reduced length but are dramatically impaired in cilia-dependent signalling functions, including growth factor and Sonic hedgehog signalling. To better understand the developmental impact of ATR loss of function, we also used zebrafish as a model. Zebrafish embryos depleted of Atr resembled ATR-SS morphology, showed a modest but statistically significant reduction in cilia length and other morphological features indicative of cilia dysfunction. Additionally, they displayed defects in left-right asymmetry including ambiguous expression of *southpaw*, incorrectly looped hearts and randomized localization of internal organs including the pancreas, features typically conferred by cilia dysfunction. Our findings reveal a novel role for ATR in cilia signalling distinct from its canonical function during replication and strengthen emerging links between cilia function and development.

## Introduction

Ataxia telangiectasia and RAD3-related protein (ATR) was the first identified causal defect for Seckel syndrome (SS) (SCKL1, MIM no. 210600), a disorder characterized by microcephaly and growth delay (1,2). ATR is the central kinase that activates DNA damage response (DDR) signalling following replication stalling or genotoxic damage (3). ATR is activated by single-stranded regions of DNA that form during replication stalling/collapse and promotes cell cycle checkpoint arrest as well as a plethora of responses that enhance recovery from replication damage. As DNA damage is encountered during most cycles of replication, ATR is essential (4–6). Patient-derived ATR-deficient SS cell lines (ATR-SS) show an impaired response to replication damage, and studies generally attribute ATR's developmental role to this function (2,6).

In contrast to ATR, many other characterized genetic defects for SS as well as those for related disorders conferring microcephalic primordial dwarfism (MPD) are found in centrosomal proteins or those that cause centrosomal defects (7). For example, microcephalic osteodysplastic primordial (MOPD)-type II, an MPD disorder, is caused by mutations in *PCNT*, which encodes the centrosomal protein, pericentrin (PCNT) (8,9). Additionally, defects in CEP152 and CENPJ also cause SS or primary microcephaly (MCPH), in which the clinical manifestation predominantly affects head size with less or no significant impact upon growth (7,10–12). Indeed, causal genetic defects for MCPH, such as *MCPH1*, *CENPJ/CPAP*, *CEP213/CDK5RAP2*, *ASPM*, *WDR62*, *CEP135*, *CEP63* and *STIL*, encode centrosome or spindle-associated proteins, providing a further link between microcephaly and centrosomal-spindle function (11,12). A proposed role for centrosomes is to regulate the symmetry of cell division, which is important during neurogenesis (11). However, the centrosome is also the basal body from which cilia are formed (13), providing an additional factor to consider for the basis underlying MPD disorders. Indeed, deficiency of PCNT also causes defects in cilia formation and function (14–16). Further, a recent study identified defects in PLK4, a protein required for centriole biogenesis, in additional patients with MPD (17).

Cilia are antenna-like structures that emanate from a modified centriole (18,19). Previously, they have been divided into primary cilia, which are mechano- and chemosensory organelles and motile cilia, which induce a fluid flow and promote motility. However, motile cilia retain some ability to sense as well as transduce molecular cues (20,21). Cilia function to transmit and receive extracellular signals and are required for signalling pathways including sonic hedgehog (Shh) and Wnt signalling (22,23). Shh signalling is important during development, including neuronal embryogenesis (24–26).

Defects in primary cilia formation or function underlie a range of developmental disorders collectively termed ‘ciliopathies’ (19). Ciliopathies are clinically heterogeneous disorders with features that include respiratory complications, renal abnormalities, retinal degeneration, structural heart defects and growth retardation. Abnormalities in neuronal development have also been reported in ciliopathies (19). Indeed, mounting evidence suggests that cilia function is required for normal neuronal development and/or growth. For example, mutations in *POC1A* and *PLK4*, which encode proteins involved in centriole function, impact upon ciliogenesis and cause primordial dwarfism (17,27). Significantly, we recently reported that defects in the origin licensing proteins, which cause Meier-Gorlin syndrome (MGS), dramatically impede ciliogenesis and cilia signalling (16).

Zebrafish have been particularly useful for characterizing the developmental impact of deficiencies in cilia formation/signalling (28,29). Such studies have revealed a role for cilia in left-right patterning (30–34). In zebrafish, primary as well as motile cilia can be readily visualized and analysed for their signalling capacity. Cilia of the left-right organizer perpetuate a counter-clockwise flow and elicit calcium spikes (35). Readouts for cilia function include the level of nodal flow, left-sided expression of nodal-related genes and cardiac looping, a measure for oriented organ morphogenesis (30,32–34).

We previously observed that ATR-SS cells display a modestly elevated frequency of supernumerary centrosomes, a phenotype shared with PCNT-deficient MOPD-type II cells and ORC-deficient MGS cells (8,16,36). Given that cilia develop from centrosomes/centrioles and the strong relationship between centrosome function and MPD, we examined whether ATR is also required for cilia formation and function. Strikingly, we show that ATR is largely dispensable for cilia formation, but has a significant role in promoting cilia-dependent signalling. Furthermore, we show that ATR deficiency in zebrafish causes an abnormality in cilia similar to that seen in human cells, but profoundly confers patterning defects reflective of ciliary dysfunction. This represents a novel and previously uncharacterized role for ATR. Finally, we provide evidence that this function of ATR is distinct to its role in promoting recovery from replication fork stalling.

## Results

### ATR-SS cells display subtle centrosome and cilia abnormalities

**Figure 1.**
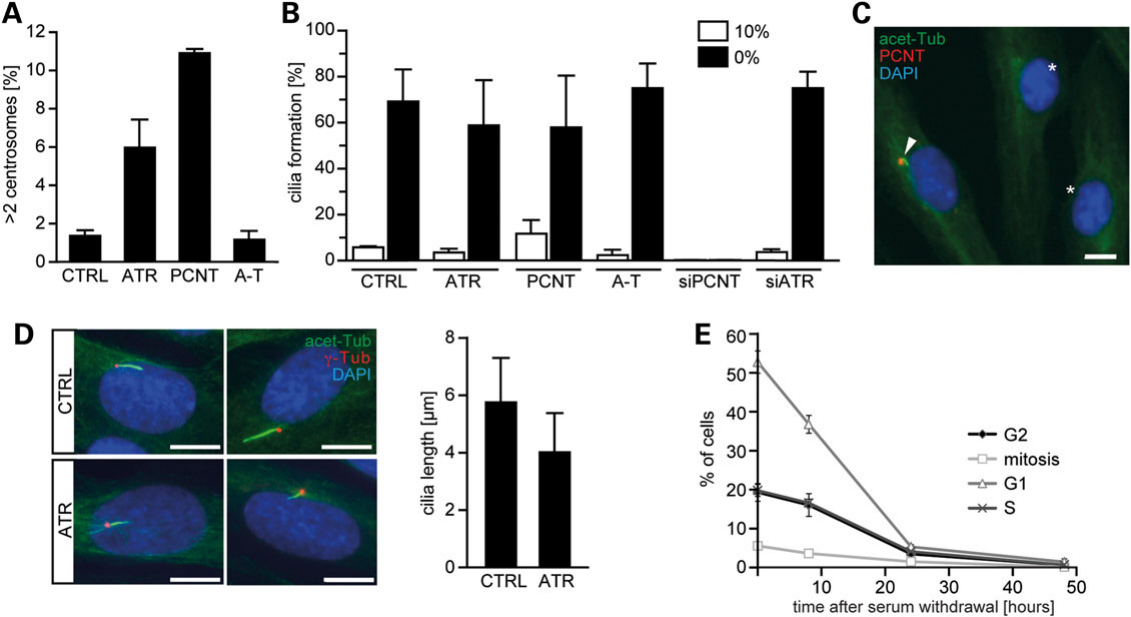
ATR-SS cells show supernumerary centrosome and shortened cilia. (**A**) Exponentially growing hTERT fibroblasts from a control (1BR3) (C), an ATR-SS patient (ATR), a PCNT-MOPD type-II patient (PCNT) or an A-T patient (A-T) were analysed for centrosome numbers following staining with anti-γ-tubulin. Cells with more than two centrosomes were scored. (**B**) Patient hTERT fibroblasts [as in (A)] or 1BR3 hTERT cells subjected to PCNT or ATR siRNA were serum-starved for 48 h (0%) or maintained in serum (10%) and processed to identify cilia using anti-acetylated tubulin and anti-γ-tubulin antibodies, which mark the entire cilia or basal body, respectively. (**C**) Typical cells lacking cilia following siRNA PCNT. The arrow shows PCNT staining (i.e. no siRNA-mediated knockdown) and a cilium; asterisks denote cells with no PCNT or cilia. (**D**) Cilia forming in ATR-SS cells are slightly shorter than those in control cells. This is evident visually (left panel) and following quantification of length (right panel). *P* < 0.01, Mann–Whitney rank-sum test. (**E**) Control cells exit the cell cycle 24 h after serum starvation. Similar kinetics of cell cycle exit were observed in ATR-SS and PCNT cells (data not shown). Cell cycle markers were: G1, P-Rb^+^; S phase, BrdU^+^; G2, CENPF-positive; mitosis, P-H3^+^. All results represent the mean ± SD of three experiments.

We previously reported that ATR-SS patient cells harbour elevated supernumerary centrosomes (36). However, in this previous study, nocodazole, which has been reported to enhance centrosome abnormalities, was added to promote accumulation of mitotic cells. Here, we examined centrosome numbers in the absence of nocodazole in fibroblasts derived from a control and an ATR-SS patient (Fig. 1A). Fibroblasts from patients with mutations in *PCNT* (PCNT) were included as a control with impaired cilia function. A-T was included as a line with a distinct defect in DDR signalling, to substantiate the specificity of the findings for ATR. A-T and ATR-SS cells have a similar growth rate (data not shown), suggesting that any differences obtained cannot be attributed to differences in proliferation. Control and A-T fibroblasts showed <1% cells with more than two centrosomes. In contrast, ATR-SS, and more markedly, PCNT-deficient MOPD-II fibroblasts, displayed an increased frequency of supernumerary centrosomes.

Cilia formation was examined 48 h after serum starvation in patient fibroblasts. Control, ATR-SS, A-T and PCNT-patient fibroblasts form cilia efficiently under this condition (Fig. 1B). Cilia also formed efficiently following siRNA-mediated depletion of ATR (*ATR* siRNA). In contrast, *PCNT* siRNA ablated cilia formation (Fig. 1B and C), consistent with findings that PCNT is required for cilia formation, but PCNT-patient cells are hypomorphic (14–16,37). Quantitative assessment of cilia length in ATR-SS cells revealed a modest but statistically significant reduction when compared with control cells (Fig. 1D) (*P* < 0.01, Mann–Whitney rank-sum test). ATR-SS cells efficiently exited the cell cycle following serum starvation (Fig. 1E).

### Cilia-dependent response to PDGF is impaired in ATR-SS cells

**Figure 2.**
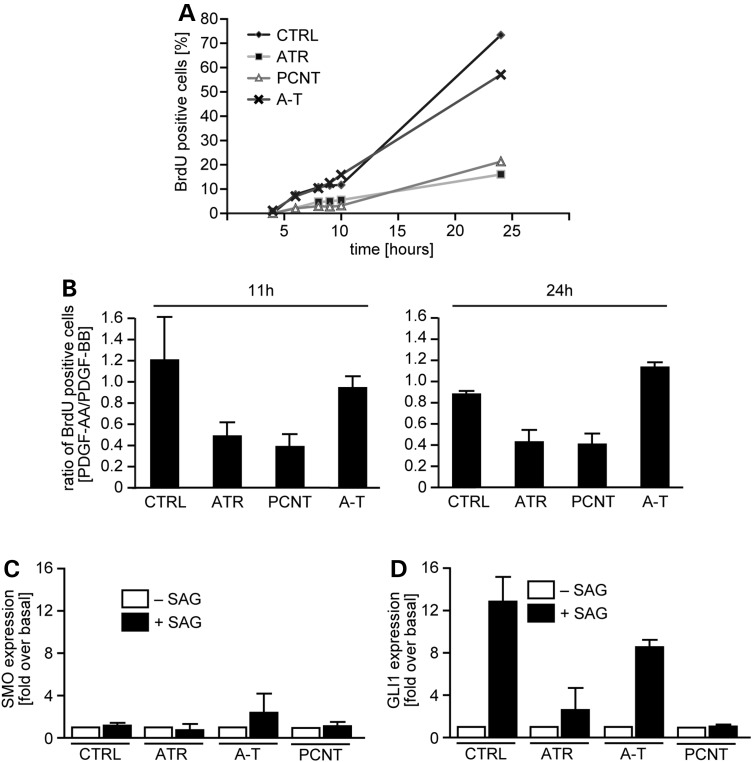
ATR-SS cells show impaired cilia-dependent growth-factor signalling. (**A**) Patient-derived hTERT fibroblasts were serum-depleted for 48 h. Following serum and BrdU addition, the percentage of BrdU^+^ S phase cells was monitored as indicated. At least 200 cells were monitored. (**B**) The indicated *hTERT* fibroblasts were serum-depleted for 48 h. PDGF-AA or -BB and BrdU were then added and the percentage of BrdU^+^ cells (i.e. cells that have entered S phase) estimated by IF 11 and 24 h later. Results are expressed as the ratio of BrdU^+^ cells following treatment with PDGF-AA versus PDGF-BB. A ratio of 1 demonstrates equal signalling from PDGF ligand isoforms (-AA or -BB) and <1 demonstrates an impaired response to PDGF-AA. The percentage of BrdU^+^ cells after PDGF-BB treatment after all siRNA treatments is shown in Supplementary Material, Figure S1. This verifies that the indicated siRNA does not impact upon PDGF-BB signalling. Results represent the mean ± SD of three experiments. (**C** and **D**) qRT–PCR analysis of SMO and GLI1 transcript levels in the indicated cells with or without SAG at 24 h. SMO is a reference as it does not change after SAG addition. The WT and PCNT data are part of a previously published data set (16). The analysis of ATR- and A-T-deficient cells was carried in the same experiments and has not been previously published. Results represent the mean ± SD of three experiments.

We examined whether ATR deficiency affects cilia function. We previously observed that S phase entry following cell cycle exit and re-entry (after serum addition) was delayed in two Sensenbrenner syndrome cells (deficient in IFT43 or WDR35), which have impaired intraflagellar transport and cilia function, and in ORC1-deficient cells, which show very delayed cilia formation (16). We proposed that cilia-dependent signalling promotes cell cycle re-entry following serum starvation. To examine ATR dependency, we monitored S phase entry by bromodeoxyuridine (BrdU) labelling following cell cycle exit (48 h in low serum) and subsequent serum addition. Strikingly, both ATR-SS and PCNT fibroblasts showed delayed S phase entry when compared with control and A-T fibroblasts (Fig. 2A). The overlapping cellular phenotype caused by IFT43, WDR35, PCNT or ATR deficiency is consistent with the notion that ATR loss may impair cilia function (16).

Platelet-derived growth factor (PDGF) signalling promotes cell cycle entry from G0 (38). Two PDGF ligand isoforms and their cognate receptors have been identified: the PDGF receptor α (PDGFR α) specifically localizes to primary cilia, is upregulated in serum-starved cells and responds to the PDGF-AA ligand isoform (39) and the PDGFRβ responds to the PDGF-BB isoform and localizes predominantly on the plasma membrane. The response to PDGF-AA represents a cilia-dependent pathway promoting cell cycle entry (16). We exploited PDGF-AA and -BB isoforms to examine cilia function as it allows parallel membrane-dependent versus cilia-dependent signalling assessment. Following serum starvation for 48 h, cells were treated with PDGF-AA or -BB isoforms for 11 or 24 h (Fig. 2B). BrdU was added and cell cycle re-entry monitored by immunofluorescence (IF) as the percentage of BrdU^+^ cells. Control and A-T fibroblasts showed a similar ratio of BrdU^+^ cells when exposed to PDGF-AA or -BB; both ATR-SS and PCNT-deficient fibroblasts showed substantially diminished BrdU^+^ cells following PDGF-AA addition (Fig. 2B).

### ATR-deficient patient cells show impaired Shh signalling

Cilia are enriched for actively signalling receptors. They thus coordinate and transduce signals required during development such as the Shh pathway (19,24–26). Cellular responses to secreted Shh are mediated by two trans-membrane proteins: patched receptor (PTCH-1) and smoothened (SMO). Shh binds to PTCH-1, alleviating suppression of SMO. SMO activation triggers GLI2 nuclear localization promoting transcriptional upregulation of Shh-pathway response genes, including *GLI1*, *PTCH1* and *HHIP.* Using quantitative reverse transcriptase–polymerase chain reaction (RT–PCR), we assessed the transcriptional activation of *GLI1*, using SMO as an internal control. Following SAG addition, an SMO-binding Shh pathway agonist, control cells showed an approximately 10-fold increase in *GLI1*, but not *SMO* transcript levels (Fig. 2C and D). A similar, slightly lower impact (∼8-fold) was observed in A-T fibroblasts. In contrast, *GLI1* transcript levels barely increased in either ATR or PCNT-deficient fibroblasts (Fig. 2C and D). This strongly suggests that ATR-deficient fibroblasts are impaired in Shh signalling, which we attribute to their abnormal cilia function.

### ATR is required for normal cilia formation and function in zebrafish

**Figure 3.**
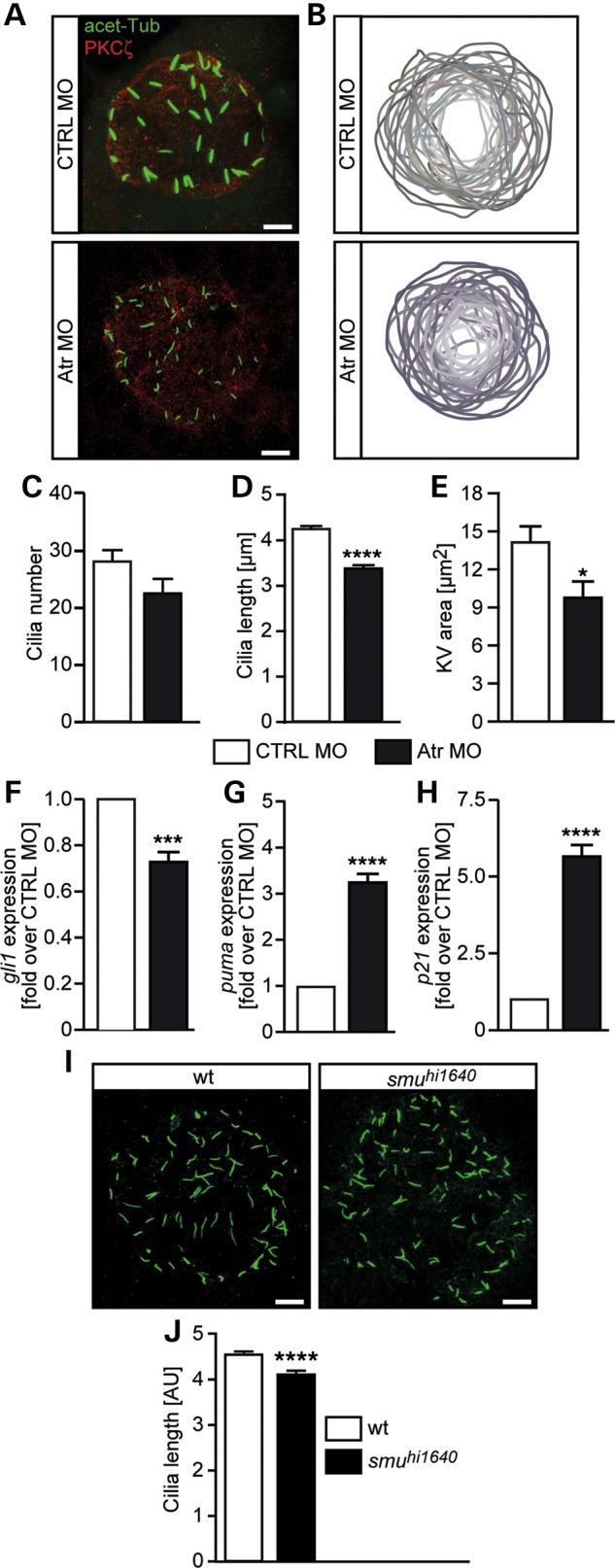
Atr depletion confers shorter cilia in zebrafish. (**A**) Representative images of cilia in the KV of zebrafish embryos. Cilia were stained with an antibody against acetylated tubulin. Outlines of the KV were visualized by counterstaining with an antibody against atypical PKC (PKCζ). Scale bars: 5 µm. (**B**) Overlay of KV outlines of CTRL MO- or Atr MO-injected embryos [eight somite stage (ss)]. Depletion of Atr results in slightly smaller KVs. (**C**) Cilia number was not significantly altered upon Atr depletion. Three independent experiments with 31 embryos per condition. *P* = 0.0770, Student's *t*-test. (**D**) KV cilia were short in embryos depleted for Atr. *n* = 7. About 10–831 cilia from 29 to 31 embryos. *P* < 0.0001, Student's *t*-test. (**E**) KV area measurements of CTRL- and Atr MO-injected embryos at eight ss. Three independent experiments with 31 embryos in total. *P* = 0.0166, Student's *t*-test. (**F**) *gli1* expression at 24 hpf after injection of either CTRL MO or Atr MO. Four independent experiments. *P* = 0.0008, Student's *t*-test. (**G**) Relative expression of *puma* in injected embryos (24 hpf). *n* = 4, *P* < 0.0001, Student's *t*-test. (**H**) p21 expression in 24 hpf embryos summarizing four independent experiments. *P* < 0.0001, Student's *t*-test. (**I**) Representative images of KV cilia in a zebrafish mutant for Smo (*smu^hi1640^*). Scale bars: 5 µm. (**J**) Average cilia length in *smu^hi1640^* mutants and wild-type littermates. *n* = 1094–1826 cilia from 20 to 27 singly genotyped embryos. *P* < 0.0001, Student's *t*-test.

To evaluate the developmental impact of ATR's role in cilia signalling, we chose zebrafish following injection with antisense morpholino oligonucleotides (MOs) as assays to monitor cilia formation and function have been well described in this system (40). First, we assessed cilia formation following *Atr* depletion*.* We depleted *Atr* using a previously published *Atr*-specific antisense MO (Atr MO) (41) and labelled cilia within the Kupffer's vesicle (KV) through staining of acetylated tubulin. The KV consists of a limited number of monociliated cells and functions as a temporal left-right organizer. Following Atr depletion, we observed that the KV area appeared slightly smaller (Fig. 3B) and the cilia modestly shorter (Fig. 3A). Quantification of cilia and KV parameters revealed that cilia formed normally and their numbers, although slightly diminished, were not significantly different from control MO-injected embryos (Fig. 3C). Strikingly, consistent with the visual images, quantitative assessment of cilia length revealed a small but highly significant shortening of cilia following Atr depletion (Fig. 3D). Quantification of the KV area substantiated this visual observation (Fig. 3E). As the cilia number was not significantly changed and because hitherto there has not been any obvious correlation between cilia length and KV area, we suggest that the smaller KV area in Atr MO embryos reflects the overall smaller embryos.

Intrigued by this result, we examined whether primary cilia were similarly affected by Atr loss. Analysis of Hedgehog pathway activity by qPCR revealed lower levels of *gli1* in Atr MO-injected embryos when compared with Ctrl MO-injected embryos (Fig. 3F). Moreover, Atr depletion significantly increased *puma* and *p21* levels (Fig. 3G and H), which have been reported to be negatively regulated by the Hedgehog pathway (42). Finally, comparison with a well-characterized mutant for *Smoothened* (*smu^hu1640^*) (43) revealed a similar reduction in the length of cilia (Fig. 3I and J). Together these findings demonstrate that the impact of Atr on cilia size is evolutionarily conserved, being observed in zebrafish as well as in human cells.

### Morphological changes caused by Atr depletion in zebrafish

**Figure 4.**
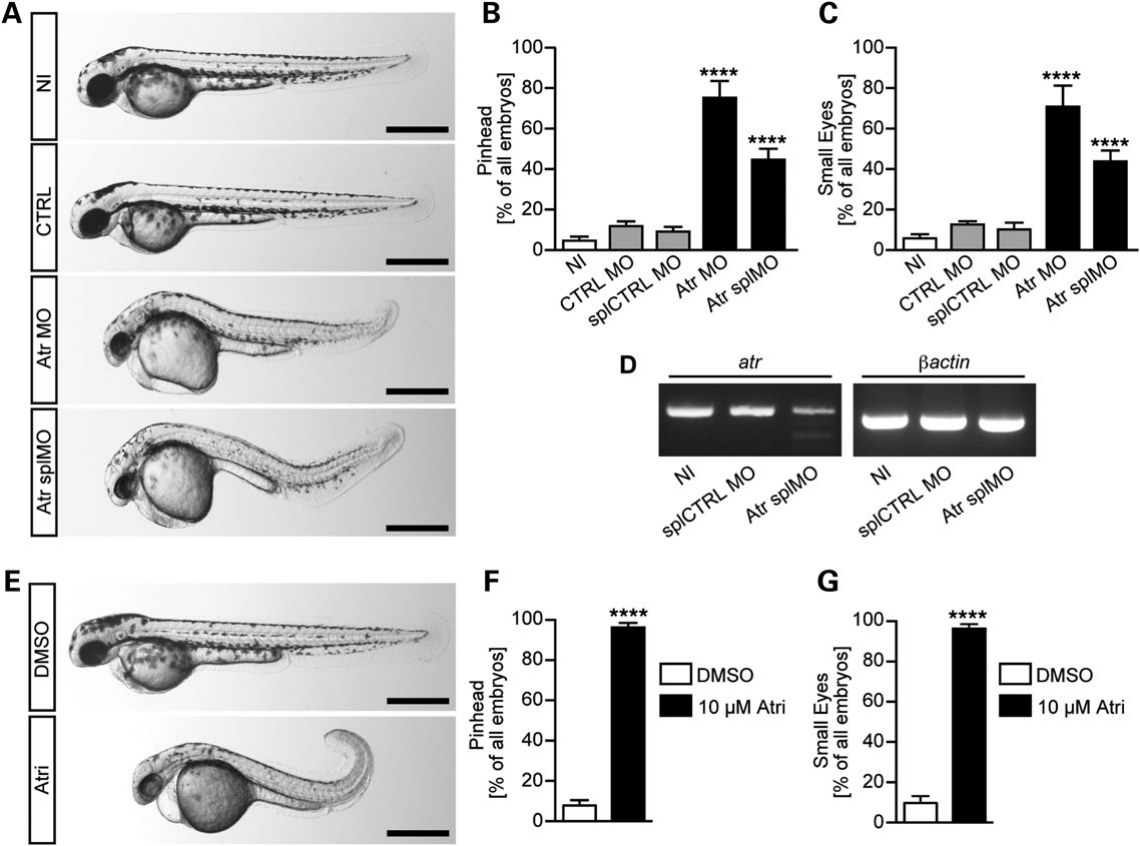
Morphological changes caused by Atr depletion in zebrafish. (**A**) Live images of 48 hpf zebrafish embryos. As controls, zebrafish were either left uninjected (NI) or control injected (CTRL) with a standard control MO (CTRL MO) or a 5 base mismatch MO (splCTRL MO), respectively. To generate Atr loss of function, injections with a translation blocking MO against Atr (Atr MO) or an MO interfering with Atr splicing (Atr splMO) were performed. Scale bars: 500 µm. (**B**) The percentage of embryos with an underdeveloped head. Graph displays mean values ± SEM, and *n* = 111–193 embryos in 5–11 experiments. ****P < 0.0001, one-way ANOVA. (**C**) Loss of Atr interferes with regular eye development. Graph displays means ± SEM, and *n* = 93–113 embryos in 5–11 experiments. ****P < 0.0001, one-way ANOVA. (**D**) Representative images of RT–PCR experiments assessing splice blocking efficiency of Atr splMO compared with controls. Left panel shows PCR of exons 2–4 of *atr* with a weaker band at the expected size and an additional smaller band representing the mis-spliced product upon Atr splMO injection. Right panel shows a PCR fragment of β*Actin* as loading control. (**E**) Live images of zebrafish embryos (48 hpf) treated with either 1% DMSO or 10 µm Atr inhibitor (ATRi-1) from the tailbud stage on. Scale bars: 500 µm. (**F**) Inhibition of Atr by chemical means (ATRi-1) results in a similar reduction of the head size of 48 hpf embryos. Graph displays mean values ± SEM, and *n* = 95–217 embryos in five experiments. *P* < 0.0001, Student's *t*-test. (**G**) The percentage of embryos developing smaller eyes upon Atr inhibition. Results show the mean values ± SEM, and *n* = 95–217 embryos in five experiments. *P* < 0.0001, Student's *t*-test.

To assess whether Atr depletion causes an SS-like phenotype in zebrafish, we examined head and eye size of Atr MO-injected embryos. Following injection with the previously characterized Atr MO or a second MO interfering with splicing of *atr* pre-mRNA (Atr splMO), we observed that a large percentage of embryos had an underdeveloped head (Fig. 4A and B). SS is furthermore characterized by a reduction of anterior neural structures and consistent with that, we observed smaller eyes in embryos depleted of Atr (Fig. 4C). As the phenotype obtained with the splice blocking MO was milder than that with the translation blocking MO, we assessed its ability to interfere with splicing. RT–PCR demonstrated that splicing of exon 3 was only partially inhibited when compared with the greater impact conferred by the translation blocking MO (Fig. 4D). Atr loss-of-function embryos also appeared smaller than control embryos, consistent with the characteristic primordial dwarfism (intrauterine and postnatal) of the patients (16).

To substantiate that these observations are a consequence of Atr deficiency, we exploited a recently generated small molecule inhibitor of Atr, ATR kinase inhibitor III (ATRi-1). To avoid interference with gastrulation movements and to selectively target KV cilia, zebrafish embryos were treated with ATRi-1 from the tailbud stage on, when the KV starts to form. Notably, this recreated the same phenotypes as Atr MO (Fig. 4E), namely, increased frequency of pinheads and small eyes (Fig. 4F and G).

Given the abnormal cilia length observed in the zebrafish, we also examined whether Atr knockdown creates known morphological changes associated with cilia dysfunction. We observed a higher frequency of pericardiac oedema, a feature often observed in zebrafish with impaired cilia (Supplementary Material, Fig. S2A–C) (44,45). Additionally, embryos depleted for functional Atr developed an increased body curvature (Supplementary Material, Fig. S2D–F), a further feature conferred by cilia dysfunction (46).

Collectively, these findings show that depletion of Atr or inhibition of Atr activity in zebrafish causes a marked impact on the development of anterior or neural structures, similar to the phenotype observed in humans. Additionally, Atr-depleted zebrafish show some morphological features typical of cilia dysfunction.

### Atr depletion causes left-right patterning defects in zebrafish

**Figure 5.**
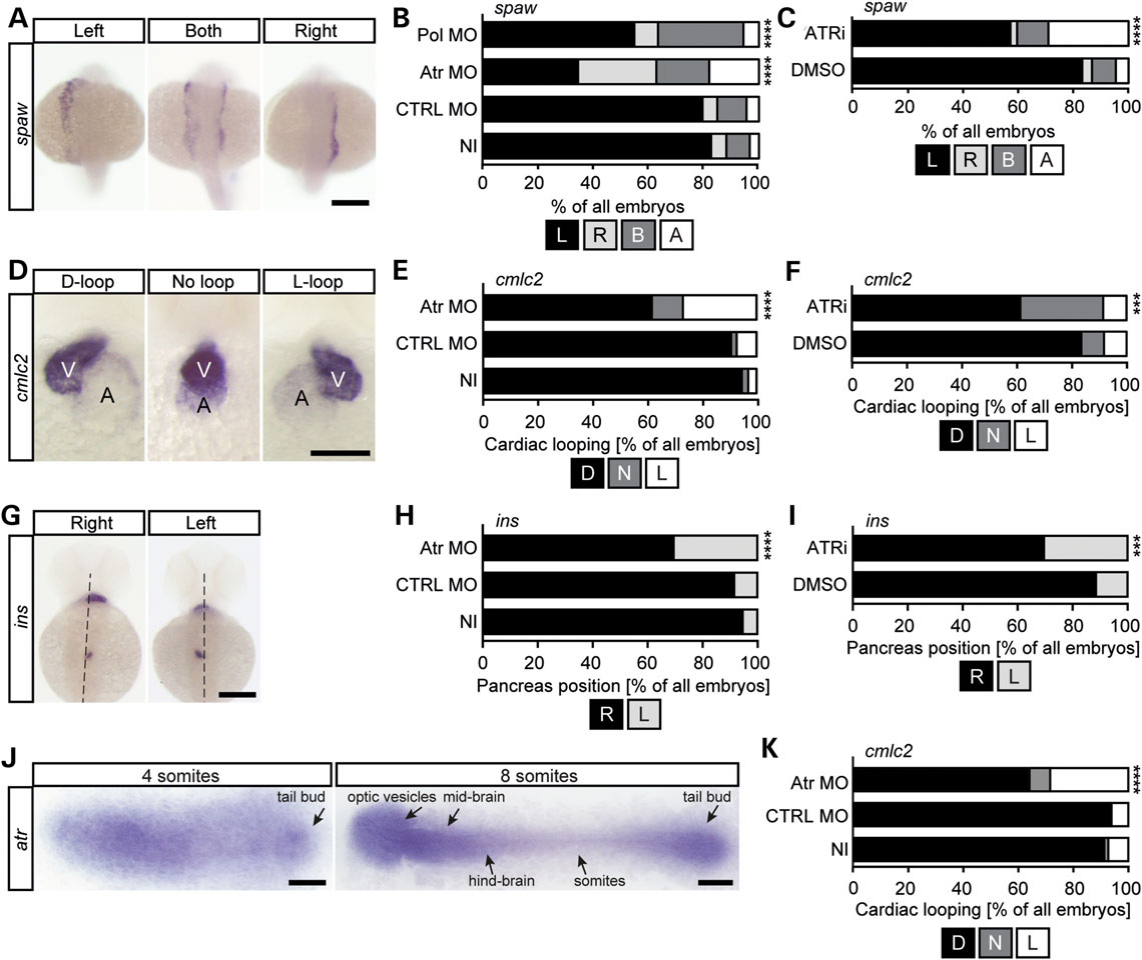
Loss of Atr impairs cilia function in zebrafish. (**A**) WMISH of 22 ss embryos illustrating *southpaw* (*spaw*) expression in the left lateral plate mesoderm (left panel). Ambiguous (middle panel) or right-sided expression (right panel) indicates aberrant left-right asymmetry. Scale bar: 200 µm. (**B**) Percentage of embryos with left-sided (L), right-sided (R), ambiguous (B) or no *spaw* expression upon Atr MO. *n*= 93–125 embryos; four experiments. *P* < 0.0001. (**C**) Chemical inhibition of Atr (ATRi-1) randomizes *spaw* expression. *n* = 124–197 embryos; six experiments. *P* < 0.0001. (**D**) Representative images of WMISH for *cardiac myosin light chain 2* (*cmlc2*) showing heart looping in 48 hpf embryos. Left panel, a correctly looped heart (D-loop); middle panel, a heart with no looping (no loop); right panel, an inversely looped heart (L-loop). A, atrium; V, ventricle. Scale bar: 100 µm. (**E**) Atr depletion impedes heart looping (48 hpf). Stacked bar graph displaying the percentage of embryos with a properly looped heart (D), an unlooped heart (N) or an inversely looped heart (L). *n* = 94–108; four experiments. *P* < 0.0001. (**F**) ATRi-1 inhibitor treatment randomizes cardiac looping similar to that observed in knockdown embryos. *n* = 106–243; seven experiments. *P* = 0.009. (**G**) WMISH for *insulin* (*ins*) labelling of pancreatic cells to assess abdominal situs development. Left panel, a dorsal view of a control. The *ins* expression domain is right of the midline (dashed line). Right panel, pancreas located left of the midline. Embryos were 48 hpf. Scale bar: 200 µm. (**H**) Stacked bar graph summarizing correct (R) or wrong-sided (L) expression of *ins* in injected embryos. *n* = 94–105 embryos; four experiments. *P* = 0.001. (**I**) Aberrant pancreas placement after Atr depletion (48 hpf). *n* = 118–245 embryos; seven experiments. *P* = 0.008. (**J**) Flat mounts of four and eight ss embryos after WMISH for *Atr*. Note the stronger staining and higher *Atr* expression in the tailbud region, where the KV forms. Anterior to the left. Scale bars: 150 µm (four ss) and 200 µm (eight ss). (**K**) KV-specific Atr knockdown randomizes cardiac looping. Stacked bar graph of embryos processed for *cmlc2* WMISH. *n* = 66–67 embryos; three experiments. *P* < 0.0001. Significance was assessed using Fisher's exact test.

To gain further insight into the consequence of Atr depletion in zebrafish, we next examined aspects of left-right patterning, which are frequently abnormal when cilia are dysfunctional. First, we assessed the expression pattern of *southpaw* (*spaw*), a nodal-related gene, which is expressed selectively in the left lateral plate mesoderm (47). When cilia function is impaired, *spaw* expression becomes randomized resulting in embryos that display right-sided or ambiguous expression (Fig. 5A). Although both uninjected and control-injected wild-type embryos predominantly expressed *spaw* on the left side, we counted a significant number of embryos with an aberrant *spaw* expression domain following Atr depletion using the translation blocking MO (Atr MO) (Fig. 5A and B) and the splice blocking MO (Atr splMO) (Supplementary Material, Fig. S3B). Rescue experiments showed further that the kinase activity of ATR is required to mediate proper cilia function as co-injection of wild-type *ATR* mRNA could partially rescue the lateralization defect in contrast to RNA encoding kinase-dead *Atr* (Supplementary Material, Fig. S3A). Finally, inhibition of Atr activity also randomized the expression of this laterality gene (Fig. 5C).

Next, we analysed heart looping after Atr depletion. Under normal conditions, zebrafish hearts undergo looping to allow the ventricle to lie left from the atrium (D-loop). Inversely looped hearts (L-loop) or hearts that fail to loop (N-loops) are commonly associated with disturbed cilia formation or function. We thus used whole-mount *in situ* hybridization (WMISH) against *cardiac myosin light chain 2* (*cmlc2*) (Fig. 5D) to visualize heart loops after Atr depletion (Atr MO and Atr splMO, respectively) or inhibition (ATRi-1). Similar to the results obtained for *spaw*, we observed a reduction of correctly looped hearts (D-loops) at the gain of irregularly looping hearts when embryos lacked functional Atr, either via Atr depletion or Atr inhibition (Fig. 5E and F and Supplementary Material, Fig. S3C).

Finally, we assessed pancreas localization through WMISH for *insulin* (Fig. 5G). This analysis demonstrated that embryos lacking functional Atr following depletion or kinase inhibition also experienced randomization of other abdominal organs as the pancreas was often located on the wrong side of the embryo (Fig. 5H and I and Supplementary Material, Fig. S3D).

These assays raise the possibility that Atr functions directly on or in cilia. We hypothesized that depletion of Atr specifically within the ciliated KV cells would lead to randomization of asymmetry development as the KV functions as a temporal left-right organizer. Consistent with this, we observed that *atr* mRNA is expressed in the tailbud region of zebrafish embryos at a time and position where the KV is found (Fig. 5J). Injection of Atr MO at the 1k cell stage to target the MO to the KV effectively interfered with normal heart looping (Fig. 5K). Collectively, these results reveal an important function of Atr in cilia biology.

### ATR is required for SMO localization in cilia

**Figure 6.**
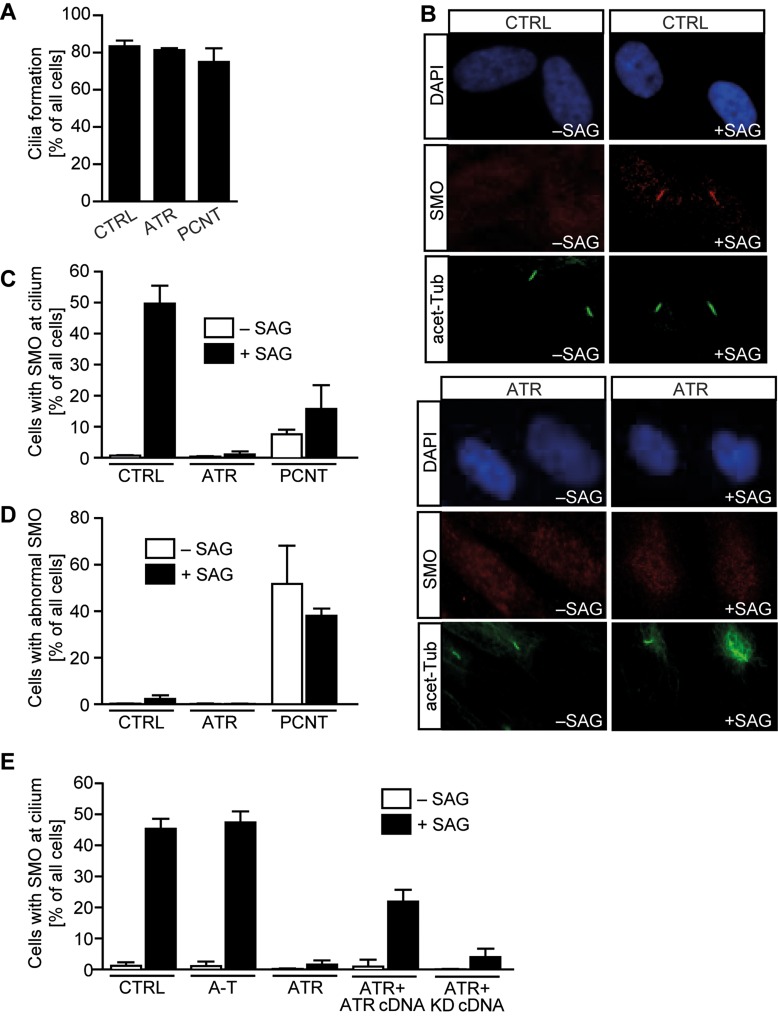
ATR-SS cells show defects in SMO recruitment to cilia and GLI1 transcript activation. Patient-derived hTERT fibroblasts were serum-depleted for 48 h. SAG was then added for 24 h. (**A**) Under this prolonged condition of serum starvation (72 h), cilia form normally in all cell lines. (**B**) In control cells, in the absence of SAG, SMO localized diffusely and not specifically at cilia. In the presence of SAG, strong uniform SMO staining is observed along the cilia length (detected using acetylated tubulin). (**C**) The percentage of cells with co-localized cilia and SMO with or without SAG treatment. (**D**) The percentage of ciliated cells with abnormally localized SMO with or without SAG treatment. (**E**) SMO localization at cilia in ATR-SS cells can be restored following expression of ATR cDNA but not kinase dead ATR cDNA (KD). Results represent the mean ± SD of three experiments. WT and PCNT data are part of the data set previously published in Stiff *et al.* (16).

The findings mentioned earlier provide strong evidence that ATR deficiency has a small impact on cilia length, but a strong impact on cilia signalling and function during development. Finally, we aimed to gain insight into how ATR affects cilia function. To examine steps during Shh signalling, we used SAG, a direct agonist of SMO. When cells are treated with SAG, SMO accumulates in the cilium (48,49). As ATR depletion did not dramatically impact upon cilia formation, we examined whether it affected SMO accumulation in cilia in the presence of SAG. At 72 h after serum starvation without SAG, we observed that ∼80% of the control fibroblasts formed cilia (Fig. 6A) with SMO localized in a diffuse pan nuclear manner (Fig. 6B). Addition of SAG for the final 24 h resulted in SMO concentration at the cilia, with ∼50% of the control cells showing co-localized acetylated tubulin and SMO (Fig. 6C). PCNT fibroblasts formed cilia at a level similar to control cells, but co-localization of SMO and acetylated tubulin was reduced and/or abnormal (Fig. 6A–D). Strikingly, ATR-SS cells showed almost no localization of SMO at the cilia either with or without SAG (Fig. 6E). Additionally, we observed that a subset of ATR-SS cells showed disordered staining and localization of acetyl tubulin following SAG addition (Fig. 6B). In summary, although SMO localized at the cilia in PCNT cells, it was frequently abnormal; in contrast, ATR-SS cells showed almost no SMO localization at the cilia, suggesting that Shh signalling depends on ATR-mediated translocation of SMO to the cilium. To substantiate a causal relationship, we verified that the defect in SMO localization could be complemented by *ATR* cDNA. Following transfection of ATR-SS cells with *ATR* cDNA, we observed substantial recovery of SMO localization to cilia (Fig. 6E). The partial recovery is likely due to the fact that only ∼50% of the hTERT immortalized fibroblasts undergo transfection. Nonetheless, the substantial recovery strongly argues that the defect is caused by defective ATR.

### ATR impact on SMO localization is replication-independent

**Figure 7.**
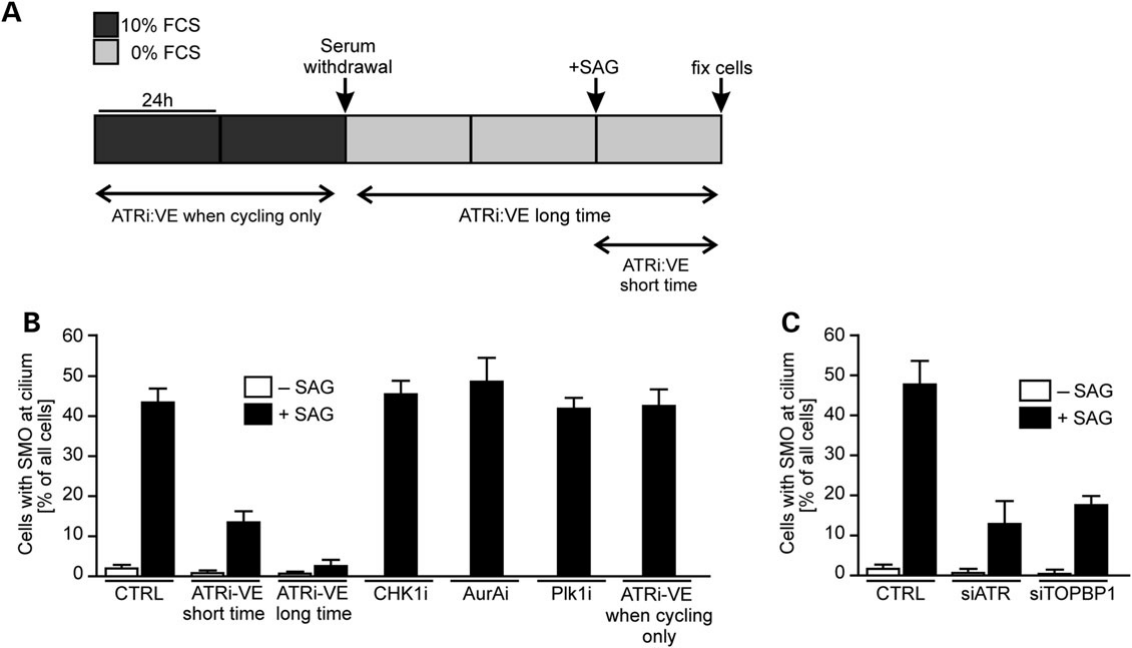
Deficiency in TOPBP1 but not inhibition of CHK1, PLK1 or Aurora A also causes defective cilia signalling. (**A**) Diagram showing the timing of ATRi:VE addition, serum withdrawal and SAG addition. (**B**) Control cells were treated with an ATR inhibitor (ATRi:VE) concurrent with serum deprivation for 72 h (long time) or deprived of serum for 48 h and ATRi:VE added for 24 h (short time). In both cases, SAG was added for the final 24 h. The percentage of cells with SMO localized at cilia was scored. Control cells were similarly treated with inhibitors against CHK1 (UCN01), Aurora A (AurAi) or PLK1 (Plk1i), concurrent with serum deprivation. For ATRi:VE when cycling, cells were treated for 48 h with the ATRi:VE before it was washed out and cells deprived of serum for 72 h as before, with SAG being added for the final 24 h. The results represent the mean ± SD of three experiments. Note that analysis of cells without SAG was not undertaken for the inhibitor experiments, as we had routinely observed that the absence of SAG led to very low numbers of cells with SMO localized at the cilia. (**C**) 1BR3 hTERT cells were treated with oligonucleotides directed against non-specific control sequences (C), ATR (siATR) or TOPBP1 (siTOPBP1). SAG was added for the final 24 h, and the percentage of cells with SMO localized at the cilia was scored as in (B).

Finally, we exploited the dependency of SMO localization in cilia on ATR to gain insight into whether ATR's role in cilia signalling is a downstream consequence of its role in promoting replication fork recovery or whether it can be uncoupled from ATR's canonical function during replication. To achieve this, we exploited another ATR-specific small molecular inhibitor that we have found works efficiently in human cells (data not shown) (ATRi:VE) to examine whether ATR's kinase activity is required for SMO localization in cilia and whether ATR's function necessitates ATR activity during replication. Two protocols were assessed (Fig. 7A). In the first, cells were allowed to enter G0 phase by serum starvation for 48 h in the presence of ATRi:VE and then SAG was added for 24 h (maintaining ATRi:VE addition) prior to the analysis of SMO localization (termed long-term ATRi:VE). In the second protocol (termed short-term ATRi:VE), cells were serum-starved for 48 h and then ATRi:VE and SAG added for 24 h prior to the analysis of SMO localization. In the first protocol, the completion of replication and entry into G0 phase occurred under the condition of ATR inhibition; in the second protocol, ATR was only inhibited during cilia formation once a non-replicating state had been reached. In control cells (CTRL), SAG addition resulted in ∼45% of the cilia showing SMO localization (Fig. 7B). Addition of ATRi:VE during cell cycle exit and SAG addition (ATRi-VE long time) reduced the percentage of cilia with SMO localization to ∼2% (Fig. 7B). Significantly, addition of ATRi:VE after cell cycle exit (ATRi-VE short time) also resulted in a marked defect in cilia with co-localized SMO (∼10%) (Fig. 7B). Although this is slightly higher than when ATRi:VE was added throughout the period, the findings nonetheless provide strong evidence that ATR inhibition after cells have exited the cell cycle can markedly reduce SMO localization, strongly suggesting that ATR's function is distinct to its direct impact on replication. To consolidate this, we also added ATRi:VE for 2 days during the cycling period; then upon serum withdrawal and during subsequent SAG addition, ATRi:VE was removed (Fig. 7A). Normal localization of SMO was observed (Fig. 7B).

To further examine the requirement for additional factors during ATR-dependent cilia signalling, we examined the role of CHK1, which plays a critical role in ATR-dependent responses at the replication fork. In marked contrast to the addition of ATRi, addition of a CHK1 inhibitor (CHK1i) using the long-term protocol (i.e. from the point of serum starvation to analysis) did not affect SMO localization (Fig. 7B). Our previous analysis has shown that such treatment dramatically impairs other ATR-dependent endpoints such as G2/M checkpoint arrest (50). As ATR has a role at the centrosome via directly or indirectly phosphorylating Aurora A kinase or Polo kinase I (PLK1), we also added inhibitors for these two kinases (AurAi or Plki) using the long-term protocol (at the start of serum starvation) and observed normal SMO localization in the presence of SAG (Fig. 7B) (51,52). Thus, ATR function in cilia signalling does not operate via any regulation of Aurora A kinase or PLK1 kinase activities.

A recent study has shown that ATR can be activated at the nuclear envelope by mechanical stress in a manner independent of DNA damage and RPA, the single-strand DNA binding protein required for ATR activation during replication (53,54). To test whether RPA is required for SMO localization, we depleted RPA by siRNA knockdown of the RPA-70 subunit in hTERT cells approaching confluency. This procedure allowed efficient knockdown of RPA in G0 phase cells without causing significant cell death. Interestingly, G0 phase cells had reduced RPA levels when compared with cycling cells, but RPA-70 siRNA resulted in a further reduction in RPA levels (Supplementary Material, Fig. S4A). SMO localization occurred normally in these cells, consolidating the notion that ATR's role in cilia function can be uncoupled from its function during replication (Supplementary Material, Fig. S4B). Finally, ATR signalling is known to require TOPBP1, which enhances ATR activation (55). Thus, we asked whether cilia signalling also requires TOPBP1. Strikingly, and in contrast to CHK1 inhibition, *TOPBP1* siRNA conferred a defect in SMO localization similar to that observed in *ATR* siRNA cells (Fig. 7C). Collectively, these findings provide strong evidence that the role of ATR in cilia signalling is not a downstream consequence of its role in replication fork stability. Indeed, ATR inhibition after cell cycle exit has a marked impact on SMO localization. Further, CHK1, a transducer kinase for ATR DNA damage signalling, which is required for ATR's role in S phase, is dispensable for cilia signalling. RPA similarly appears to be dispensable for ATR's role in cilia signalling. In marked contrast, TOPBP1, which facilitates ATR activation, is required for SMO recruitment to cilia, consistent with the requirement for ATR kinase activity.

## Discussion

ATR has a canonical function in the DDR with an essential role in promoting recovery from stalled/collapsed replication forks. Mutations in *ATR* have been identified in a subclass of SS patients, with microcephaly and severe growth delay being the pronounced phenotypes. These clinical manifestations have been attributed to the canonical function of ATR in regulating the DDR during replication (2,6). Although this is a plausible model, other disorders with associated microcephaly and growth delay have mutations in genes required for centrosome function rather than for the DDR/replication (7). Provocatively, we previously reported that ATR-SS cells have supernumerary centrosomes. Previous studies have demonstrated a link between abnormal centrosome numbers and cilia dysfunction (13,15,27). These findings prompted us to examine whether ATR might also be required for cilia signalling. Here, we provide strong evidence that depletion or inhibition of ATR in human cells results in a subtle defect in cilia length and a more pronounced defect in cilia signalling, including Shh signalling. We demonstrate that this role of ATR is conserved in zebrafish and use this model organism to probe for a functional impact. Strikingly, zebrafish depleted for Atr display typical defects in morphology and lateralization determination, similar to that found following depletion of several other factors required for cilia formation or signalling. Collectively, these findings provide strong evidence that ATR is required for efficient cilia function during development.

Significantly, ATR deficiency in patients confers marked microcephaly, reflecting a role for ATR during neuronal embryogenesis, and additional abnormalities in growth and skeletogenesis (2,56). The link between centrosomal function and MPD disorders has been previously highlighted (11,12). Many proteins with centrosomal functions are causal for MPD disorders (7). Of these disorders, MOPD-II and MGS also have aberrant cilia signalling. Further, a recent study also showed that mutations in *MRE11*, *ZNF423* and *CEP164*, genes which encode proteins that function within the DDR, can cause nephronophthisis, providing another link between DDR signalling and ciliopathies (57). Recent patients with mutations in *MRE11* have also been reported to have microcephaly (58). Additionally, chondrogenesis involves cilia-dependent signalling (59), and ATR-SS patients and mouse models of impaired Atr function have skeletal abnormalities (6). Interestingly, one subclass of Jeune asphyxiating thoracic dystrophy patients with the same missense mutation in CEP120, a centriolar protein, have a range of skeletal abnormalities (60). The patient cells display centriole abnormalities and reduced cilia formation. For ATR deficiency, it could be the role in cilia signalling rather than, or in addition to, its canonical function in DDR signalling that confers the clinical manifestation. Importantly, embryonic neuronal development involves Shh signalling (24–26).

A marked phenotype of impaired cilia function in zebrafish is a left-right patterning defect. Strikingly, this phenotype is also observed following depletion of Atr in zebrafish, in which we detected asymmetry defects at different stages and in different organs. These phenotypes, however, have not been described as marked features of the ATR-deficient patients described to date. Furthermore, although PCNT and ORC1 deficiency also confer significant defects in cilia signalling, ATR-SS, MOPD-II nor MGS patients show the commonly described manifestations of defective ciliogenesis (e.g. renal cystic disease). However, the mutations in all of these patients are hypomorphic as *ATR*, *PCNT* and *ORC1* are essential and in each case the mutations must allow sufficient residual protein function to enable embryogenesis and patient viability. Further, ciliopathies are clinically heterogeneous and cilia dysfunction manifests in distinct ways. In this context, we note that ATR-, PCNT- and ORC1-deficient patients display skeletal abnormalities, and there is mounting evidence that cilia function during skeletogenesis, and at least one ATR-SS patient, has been described with multiple liver cysts indicative of Caroli's disease (56). Furthermore, we note that ATR deficiency was recently reported to cause retinal photoreceptor degeneration in mice, a process linked to cilia dysfunction (61).

A role in cilia function represents a novel role for ATR. Further, we provide evidence that this is distinct to ATR's canonical role in recovery from replication fork stalling as diminished cilia signalling is observed when ATR is inhibited after the cessation of replication and as the process is not dependent on CHK1, an essential protein required for recovery from replication fork stalling. Our findings, moreover, cannot readily be attributed to any changes in growth rate. However, ATR's role in cilia signalling does require its kinase activity as well as TOPBP1, a protein that promotes ATR's ability to phosphorylate its substrates. Although it is difficult to entirely separate ATR's role as a DDR protein from its function in cilia signalling, the latter function does not necessitate treatment with exogenous DNA-damaging agents. However, whether DNA damage arises during cilia formation is unknown.

Our study, therefore, raises the question of the basis underlying the role of ATR in cilia signalling. Although centrosomes are required for ciliogenesis, the cilia defect need not be a direct consequence of impaired centrosome function but may reflect dual roles for the proteins (27). Nonetheless, ATR does have a function at the centrosome, where it can regulate the phosphorylation of the kinases, PLK1 and Aurora A, albeit in a damage-dependent manner (51,52,62). We tentatively propose, therefore, that ATR can be activated in a manner that does not require exogenous DNA damage or replication-dependent damage at either the centrosome or the cilia. In this context, a significant recent study showed that ATR could be activated at the nuclear envelope by forces such as mechanical stretching (53). Indeed, the findings strongly suggested that signals from the cell membrane could be transmitted to the nuclear membrane. The formation of cilia is likely to impose such a force, providing a route to activate ATR. ATR has multiple substrates that could impact upon cilia signalling. However, further studies are required to untangle the precise mechanism.

In summary, we demonstrate a novel function for ATR in cilia-dependent signalling in both human cells and zebrafish. Our findings provocatively raise the possibility that ATR-SS may represent a ciliopathy disorder and that impaired cilia function may contribute to their clinical manifestation.

## Materials and Methods

### Cell culture

hTERT-immortalized fibroblasts were control (1BR3hTERT), ATR (FO2-98hTERT), ATM (AT1BRhTERT) and PCNT (ASBhTERT). siRNA was carried out using the appropriate Smartpool (Dharmacon, Lafayette, CO, USA) and Metafectene Transfection Reagent (Biontex, Munich, Germany). The ATR inhibitor, VE-821 (Selleckchem/Stratech, Newmarket, UK), was used at 10 µm. The Aurora A kinase inhibitor, MLN8237 (Selleckchem/Stratech), was used at 0.5 µm. The Polo-like kinase 1 inhibitor, BI2536 (Axon Medchem, Groningen, The Netherlands), was used at 100 nm. The Chk1 inhibitor, UCN-01 (Merck, Kenilworth, NJ, USA), was used at 600 nm.

### Zebrafish manipulation

Zebrafish eggs were collected from natural matings and were allowed to develop up to the desired stages at 28.5°C. For knockdown studies, fertilized eggs were injected in the yolk at the 1–2 cell stage or the 1000 cell stage to target dorsal forerunner cells. Antisense MOs used were the standard control MO and a translation blocking MO for Atr, which had been described before (41). In addition, MOs targeting the boundary of intron 2 and exon 3 (Atr splMO: 5′-CAGAGCAACTACAATGTGATAGAGA) and a corresponding 5 bp mismatch control MO (splCTRL MO: CAcAcCAAgTACAATcTcATAGAGA) were used. All MOs were purchased from Gene Tools Inc. (Philomath). Rescue experiments were performed by co-injection of capped RNA encoding human ATR and a kinase dead version of human ATR (p.D2494E), respectively (plasmids were a gift from Dr A.M. Carr). Capped RNA was prepared with the T7 mMessage mMachine Kit (Ambion) using linearized plasmids. Drug treatment with either dimethyl sulphoxide (DMSO) (Sigma-Aldrich, St Louis, MO, USA) or the ATR Kinase Inhibitor III (CAS 1345675-02-7 Calbiochem/EMD Millipore, Watford, UK) was performed in egg water from tailbud stage on. All procedures on zebrafish adhered to current law and were approved by the local authority in Ulm, Germany.

### Splice blocking efficiency test (RT–PCR)

Splice blocking efficiency was tested using RT–PCR on RNA prepared from 24 h post fertilization (hpf) embryos with primers spanning over exon 3: Atr Fw: 5′-GGTTGAGTACAACCAGGCAGT; Atr Rev: 5′-AACAGGCTAAACACGACTGGA. This produces a 378 bp fragment under control conditions. When splicing is blocked and exon 3 is deleted, a PCR product of 225 bp is expected. To control for loading, a PCR for β-actin was performed with the following primers: β-actin Fw: 5′-GACATCAAGGAGAAGCTGTGC; β-actin Rev: 5′-CACTTCCTGTGAACGATGGAT.

### IF analysis

Cells grown on cover slips were fixed with 3% formaldehyde for 7 min and then with ice cold 70% MeOH for 1 min and permeabilized in 0.5% Triton X-100. For BrdU staining, DNA was denatured in 2 N HCl for 30 min. After antibody treatment and staining with 4,6-diamidino-2-phenylindole, cover slips were mounted in Vectashield mounting medium (Vector Laboratories, Burlingame, CA, USA). Samples were incubated with primary antibodies for BrdU (BU20A), CenPF, PCNT, phospho-H3 (Santa Cruz, Santa Cruz, CA, USA), γ-tubulin, acetylated tubulin (Sigma, St Louis, MO, USA), phospho-Rb (Cell Signaling, Beverley, MA, USA) and Smoothened (Abcam, Cambridge, UK). Secondary antibodies were from Sigma.

Fibroblasts were grown to 70–80% confluency followed by serum starvation in MEM containing 0.1% fetal calf serum (FCS) for 2–3 days to promote entry into G0. Cells were processed for IF as mentioned earlier and cilia visualized with anti-acetylated tubulin and γ-tubulin antibodies.

### Shh pathway assay

Fibroblasts were serum-starved for 2–3 days in MEM containing 0.1% FCS. An aliquot of 1 µm SAG (Smoothened Agonist, Calbiochem, Billerica, MA, USA) was added for a further 24 h. Cells were processed for IF as above. Cilia or the basal body was identified by antibodies against acetylated tubulin and γ-tubulin and then Smoothened staining at the cilium was assessed.

### Cilia function assay

Fibroblasts were serum-starved for 2–3 days in MEM containing 0.1% FCS. Then MEM with FCS or with 50 ng/ml PDGF-AA, PDGF-AB or PDGF-BB (Sigma) was added. S phase cells were identified by labelling with 10 µm BrdU (Becton Dickinson, Franklin Lakes, NJ, USA) and processed for IF as mentioned earlier.

### siRNA knockdown

The protocol for siRNA-mediated knockdown of PCNT and ATR was as described previously (8,63). These references include an analysis of knockdown efficiency using an identical procedure, and a similar level of knockdown was obtained in this study. RPA knockdown of RPA-70 was achieved using an RNAi SMARTpool from Dharmacon. Knockdown efficiency for RPA-70 was assessed by western blot using mouse-anti-RPA (Calbiochem cat. no. NA13) and mouse-anti-β-actin (Abcam cat. no. Ab40864).

### qPCR analysis

qRT–PCR for Smoothened and Gli1 analysis was carried out using the QuantiFast SYBR Green PCR Kit (Qiagen, Venlo, The Netherlands). SDHA was used for internal normalization, and the *Pfaffl* analysis method was applied*.* The primer sets used were Qiagen Quantitect primers for SMO and SDHA (QT00050701 and QT00059486) and for GLI1: (F) CCAGCCAGAGAGACCAACA and (R) ATCCGACAGAGGTGAGATGG. In brief, reactions containing 12.5 µl SYBR Green PCR Master Mix (Qiagen), 2.5 µl 10× Primer assay mix, 5 µl RNAse-free water and 5 µl template cDNA to a final volume of 25 µl were prepared in duplicate. Cycling was carried out using the Stratagene Mx3005P QPCR System. Cycling conditions were: 95°C for 5 min, followed by 40 cycles of 95°C for 10 s and 60°C for 30 s. Reactions were then heated to 95°C for 1 min and incubated at 55°C for 30 s.

qPCR analysis of zebrafish was performed as described previously (45) using the Absolute qPCR ROX Mix (Thermo Scientific, UK) and the Roche Universal Probe System (Roche Applied Science, Mannheim, Germany) in a Lightcycler 480 instrument (Roche). All levels were normalized to *beta-2-microglobulin* (*b2m*) (GenBank no. BC062841.1). The following primers and probes (UP) were used: *gli1* Fw: 5′-GGTCTCGATGCCAGTGGA, *gli1* Rev: 5′-CACTGACGGAGCCAGTCC, UP no. 5; *puma* Fw: 5′-CGAGATGAACGCTGTCTTCC, *puma* Rev: 5′-CCTCTCCAGTTCTGCCAGTG, UP no. 6; *p21* Fw: 5′-AAGCGCAAACAGACCAACAT, *p21* Rev: 5′-TCAGCTACTGGCCGGATTT, UP no. 142; *b2m* Fw: 5′-ACATCACTGTACAGGGGAAAGTC, *b2m* Rev: 5′-TCCGTTCTTCAGCAGTTCAA, UP no. 65.

### Centrosome analysis

Cycling fibroblasts were processed for IF as mentioned earlier and centrosomes visualized with anti-γ-tubulin and PCNT antibodies.

### Microscopy

Living zebrafish and those processed by WMISH were imaged using a Leica M125 upright microscope equipped with a Leica IC80 HD camera. KVs and cilia were assessed by confocal microscopy with a Leica TCS SP5II confocal microscope. Cilia length in *z*-stacks was measured with the help of ImageJ (64).

### WMISH

WMISH was performed according to standard protocols (65). The DIG-labelled probes for *cmlc2*, *ins* and *spaw* have been described before (45). The probe for *atr* was generated by *in vitro* transcription from a linearized plasmid containing a 1001 bp fragment of the Atr coding sequence (GenBank no. XM_691071).

### IF analysis for zebrafish work

IF analysis was as described earlier. Zebrafish cilia were labelled according to the protocol of Jaffe *et al*. (66) using a mouse-anti-acetylated tubulin antibody (1:500, clone 6-11B-1, Sigma-Aldrich) and an Alexa 488-labelled secondary antibody. KV outlines were visualized through immunostaining with an antibody against atypical PKC (1:500, clone C-20, Santa Cruz Biotechnologies).

### Statistical analysis

All statistical analysis was performed using GraphPad Prism 6. When three conditions were analysed, a one-way analysis of variance with a Bonferroni post-test was applied. Analyses including only two experimental conditions (i.e. DMSO versus Atri) were scrutinized with one- or two-tailed Student's *t*-tests. Stacked bar graphs were tested for statistical significance using a Fisher's exact test.

## Supplementary Material

Supplementary Material is available at *HMG* online.

## Authors’ Contribution

T.S., M.P., M.O.'D. and P.A.J. conceived the study. T.S., T.C.T. and M.P. did the laboratory work. M.P., M.O.'D. and P.A.J. wrote the manuscript.

## Funding

All data are provided in full in the results section of this paper or in the supplementary material. The PAJ laboratory was supported by Medical Research Council Grants (G000050 and G0500897) for this work. The M.O.'D. laboratory is supported by the Cancer Research UK and the Medical Research Council. The M.P. laboratory is supported by grants from the German Research Foundation (DFG, PH 144-1) and the Boehringer Ingelheim Ulm University BioCenter. T.C.T. is a fellow of the International Graduate School in Molecular Medicine at Ulm University. Funding to pay the Open Access publication charges for this article was provided by the Medical Research Council, grant numbers, G000050 and G0500897.

## Supplementary Material

Supplementary Data
