# Supplementary material for: ATR promotes cilia signalling: links to developmental impacts
Source: Hum Mol Genet. 2016 Feb 11;25(8):1574–87. doi: 10.1093/hmg/ddw034 (PMC4805311; doi:10.1093/hmg/ddw034)
Supplement: Supplementary Data [file supp_25_8_1574__index.html]

ATR promotes cilia signalling; links to developmental impacts — ATR promotes cilia signalling: links to developmental impacts — ATR promotes cilia signalling: links to developmental impacts — Supplementary Data 

# ATR promotes cilia signalling: links to developmental impacts

## Supplementary Data

Supplementary Data

- Supplementary Data - Docx file
