# Supplementary material for: ATR promotes cilia signalling: links to developmental impacts
Source: Hum Mol Genet. 2016 Feb 11;25(8):1574–87. doi: 10.1093/hmg/ddw034 (PMC4805311; doi:10.1093/hmg/ddw034)
Supplement: Supplementary Data [file supp_ddw034_ddw034supp.docx]

**Supplementary figure legends**

**
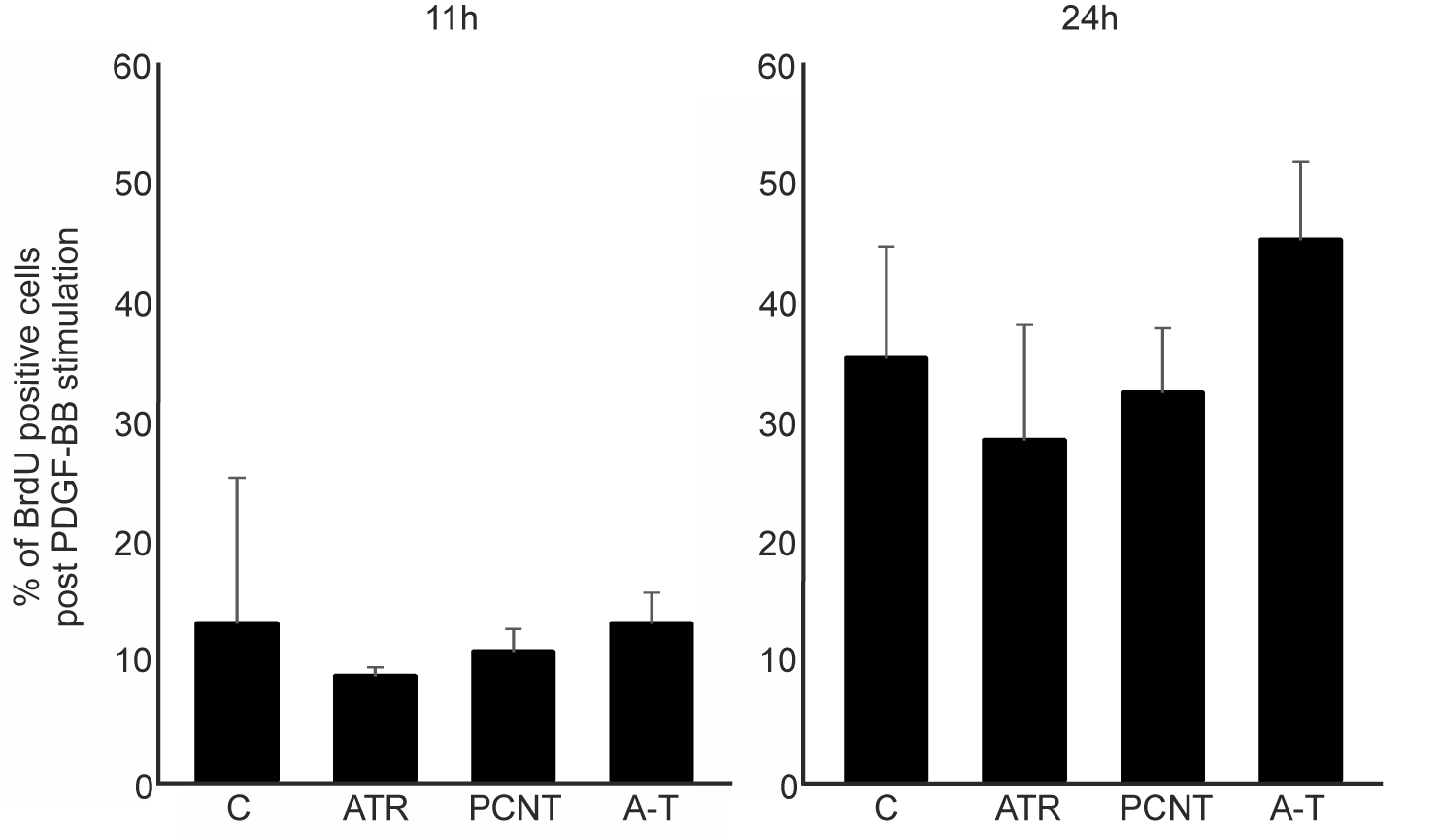
**

**Figure S1. Control and patient cells respond similarly to PDGF-BB**

The indicated *hTERT* fibroblasts were serum depleted for 48 h. PDGF–BB and BrdU was then added and the % of BrdU^+^ cells (i.e. cells that have entered S phase) estimated by immunofluorescence 11 and 24 h later. All cells respond similarly to PDGF-BB despite showing a differential response to PDGF-AA (fig 2b). Results represent the mean +/- SD of 3 experiments.


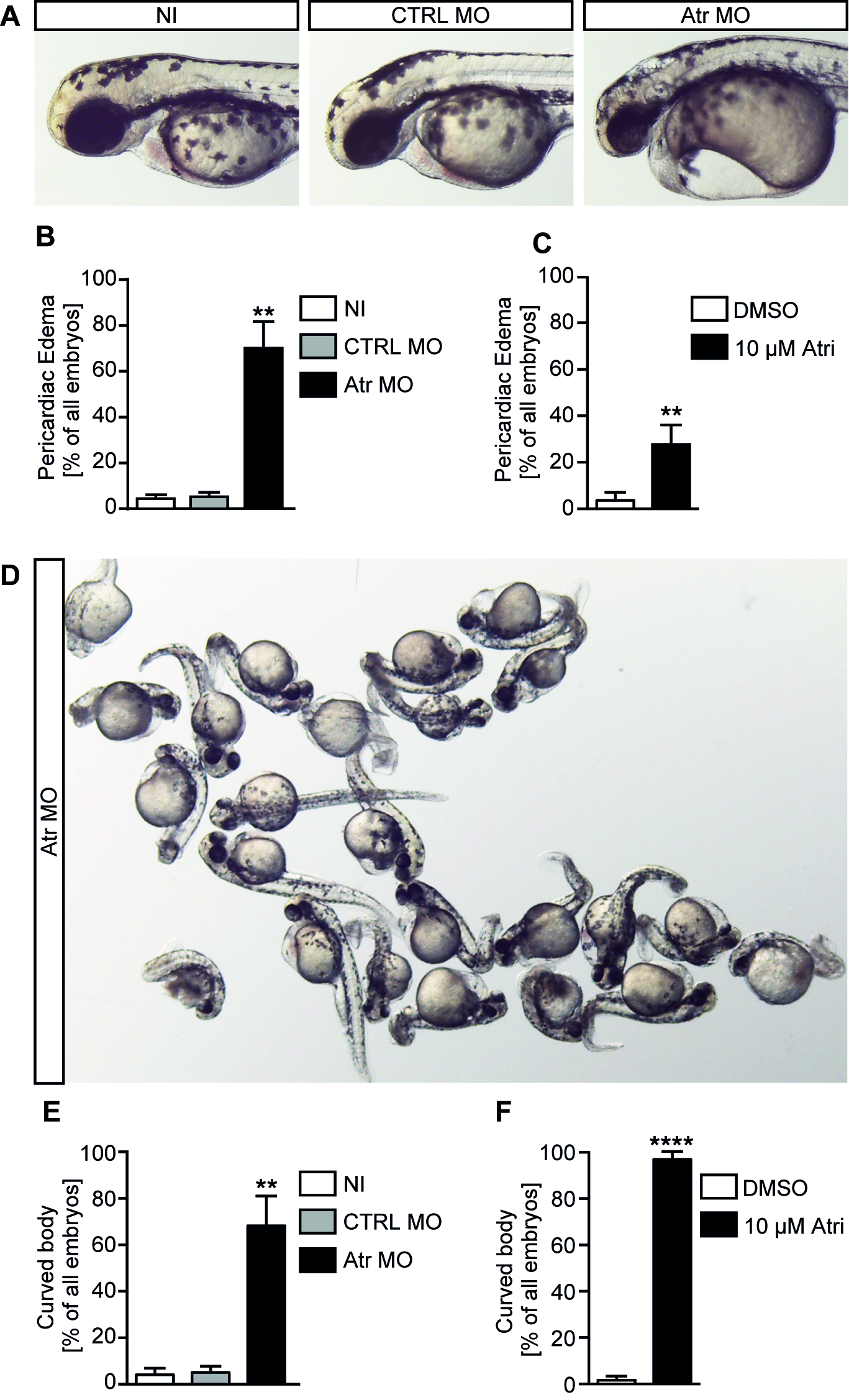


**Figure S2. Atr morphant zebrafish display morphological phenotypes typical of cilia dysfunction.**

a) Live images of 48 hpf zebrafish embryos showing pericardiac edema upon Atr knockdown. Zebrafish were either left uninjected (NI) or control injected with a standard control MO (CTRL MO) or the translation blocking Atr MO.

B) Percentage of embryos developing pericardiac edema. Graph displays mean values ± SEM, n=79-85 embryos in 3 experiments. ** indicates p<0.01, One way ANOVA.

C) Treatment with the ATR inhibitor from tailbud stage causes pericardiac edema, too. Graph displays means ± SEM, n=55-60 embryos in 3 experiments. p=0.0025, Student’s t-test.

D) Live image of a clutch of 48 hpf embryos that had been injected with Atr MO at the one cell stage. Note the large number of embryos displaying an increased body curvature, which is an indicator of a cilia dysfunction.

E) Percentage of embryos with an abnormally curved body. Graph displays mean values ± SEM, n=79-85 embryos in 3 experiments. ** indicates p<0.01, One way ANOVA.

F) Chemical inhibition of Atr (Atri) robustly leads to curved embryos. Results show the mean values ± SEM, n=55-60 embryos in 3 experiments. p<0.0001, Student’s t-test.

**
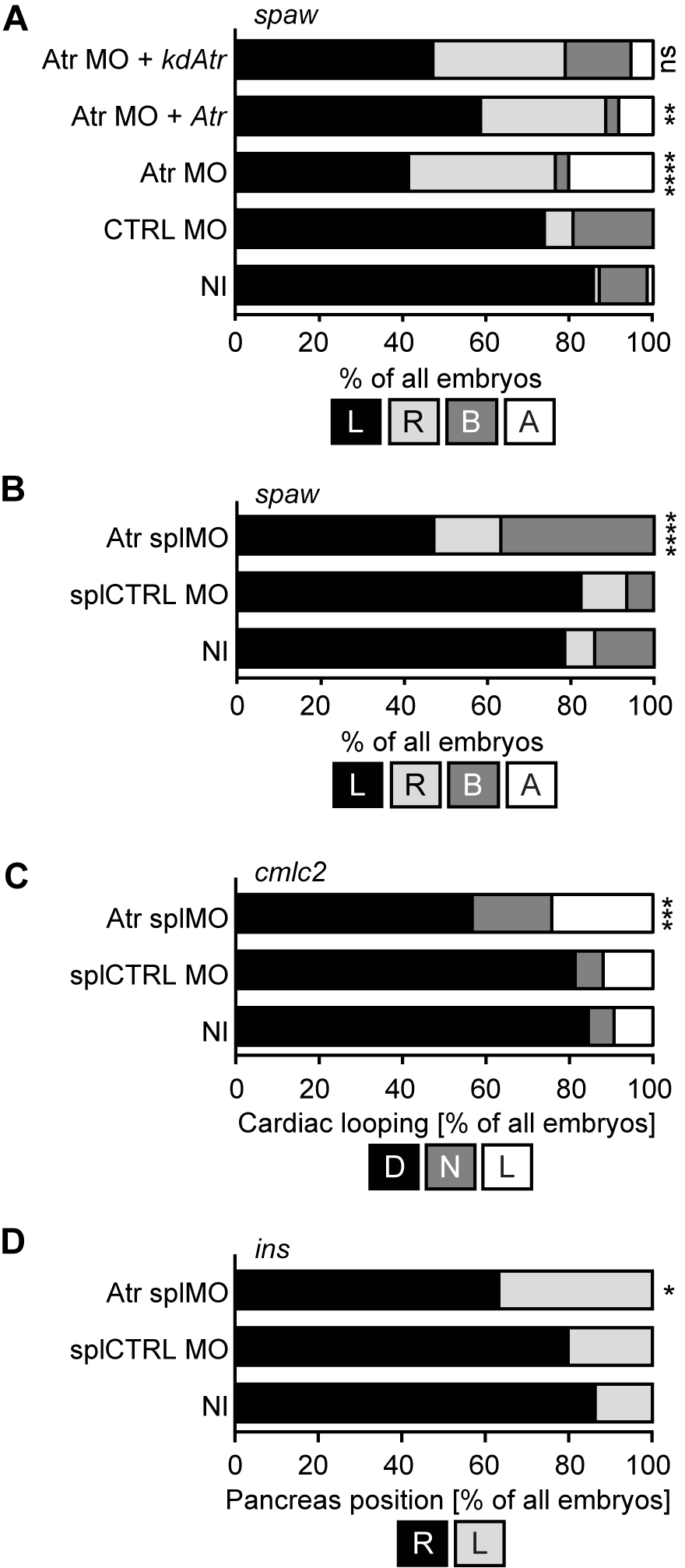
**

**Figure S3. The Atr phenotype in zebrafish can be reproduced with a second MO and rescued using human ATR.**

A) *Spaw* localisation can be partially rescued by co-injection of human ATR RNA, but not by kinase dead ATR. Stacked bar graph displaying the percentage of embryos with left-sided (L), right-sided (R), ambiguous (B) or no *spaw* expression (A). n=4 experiments with 70-97 embryos. **** indicates p< 0.0001, ** indicates p=0.0085, ns indicates p=0.2214 (Fisher’s exact test).

B) Stacked bar graph summarizing *spaw* expression pattern following injection of a splice blocking MO targeted against Atr. n= 87-98 embryos in 4 independent experiments. p< 0.0001, Fisher’s exact test.

C) Heart looping is randomized upon injection of the Atr splMO. Stacked bar graph displays the percentage of embryos with a properly looped heart (D), with an unlooped heart (N) or with an inversely looped heart (L). n= 129-136 embryos in 6 independent experiments. p= 0.0004, Fisher’s exact test.

D) Injection of a splice blocking MO significantly randomizes pancreas placement. R, *ins* expression right from the midline, L, *ins* expression left from the midline. 6 independent experiments with 132-147 embryos in total are summarized. P=0.0118, Fisher-s exact test.


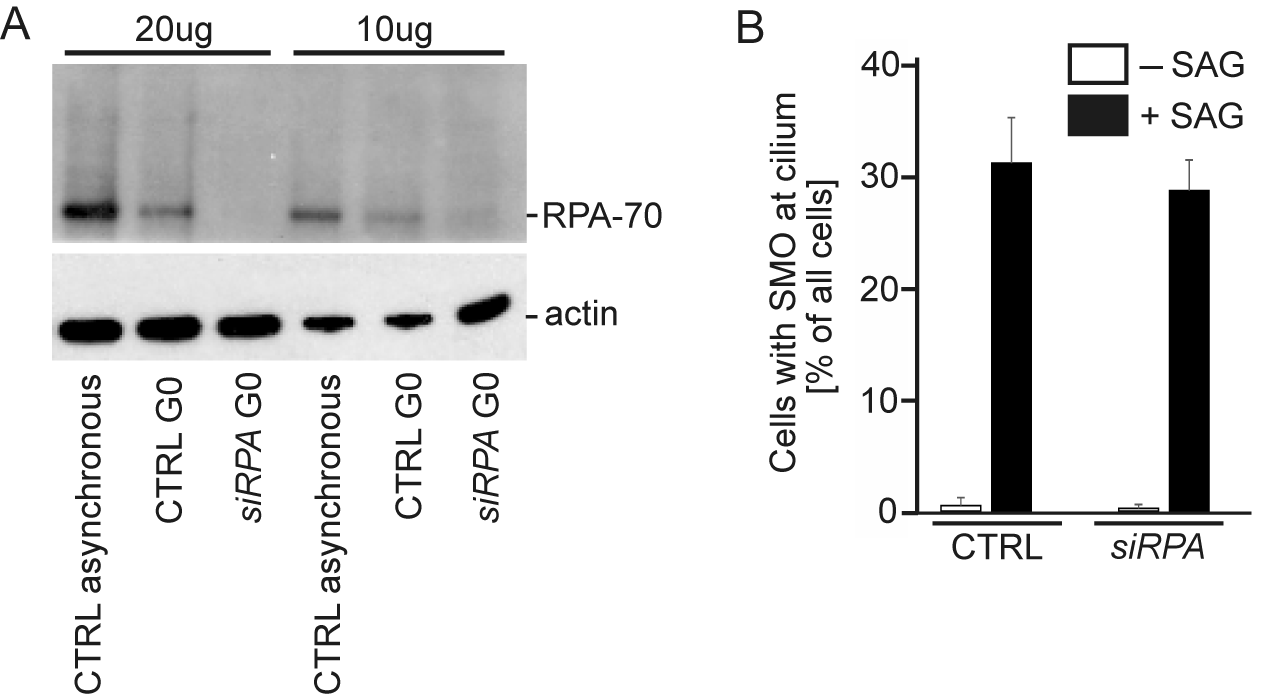


**Figure S4. RPA depletion does not affect smoothened recruitment to cilia.**

A) 1BR3hTERT fibroblasts at approximately 70-80% confluency were serum starved and treated with oligonucleotides directed against non-specific control sequences (CTRL) or RPA-70 (RPA) (G0 cells). Cells were also treated with the same oligonucleotides when at ~ 50 % confluency without serum starvation (asynchronous cells). After 3 days, cell extracts were prepared and examined by Western Blotting using anti-RPA-70 antibody to assess knockdown efficiency.

B) Following serum starvation and treatment with the siRNA oligonucleotides described above, 1BR3 hTERT cells were incubated for 3 days. SAG was added for the final 24 h and % cells with SMO localised at the cilia were scored. In the absence of SAG, SMO localised diffusely and not specifically at cilia. In the presence of SAG, strong uniform SMO staining is observed along the cilia length (detected using acetylated-tubulin). A similar response to SAG was observed in control and RPA depleted cells.

siRNA was carried out using the appropriate Smartpool (Dharmacon, Lafayette, Colorado, U.S.) and Metafectene Transfection Reagent (Biontex, Munich, Germany).
